# Hypocoagulability in Children With Decompensated Chronic Liver Disease and Sepsis: Assessment by Thromboelastography

**DOI:** 10.1097/PG9.0000000000000324

**Published:** 2023-06-09

**Authors:** Vignesh Vinayagamoorthy, Anshu Srivastava, Indranil Das, Anupam Verma, Prabhakar Mishra, Moinak Sen Sarma, Ujjal Poddar, Surender Kumar Yachha

**Affiliations:** From the * Department of Paediatric Gastroenterology, Sanjay Gandhi Postgraduate Institute of Medical Sciences, Lucknow, Uttar Pradesh, India; † Department of Transfusion Medicine, Sanjay Gandhi Postgraduate Institute of Medical Sciences, Lucknow, Uttar Pradesh, India; ‡ Department of Biostatistics and Health Informatics, Sanjay Gandhi Postgraduate Institute of Medical Sciences, Lucknow, Uttar Pradesh, India.

**Keywords:** thromboelastography, children, decompensation, chronic liver disease, coagulopathy

## Abstract

**Objective:**

To evaluate the coagulation status of children with decompensated chronic liver disease (DCLD) and infection and factors affecting it using thromboelastography (TEG).

**Methods:**

Coagulation status of children admitted with DCLD and infection was assessed by international normalized ratio (INR), platelet count, and TEG [reaction time (R), kinetic time (K), α-angle (AA), maximum amplitude (MA), coagulation index (CI), and lysis index (LY30)] at admission and at 7–14 days after treatment. CI < −3 represents hypocoagulable state. Clinical profile including systemic inflammatory response syndrome (SIRS), infection severity, bleeding, treatment response, and outcome were noted.

**Results:**

Thirty children (21 boys, median (IQR) age 78 [15.7–180] months) were studied prospectively. At admission, 29 (96.7%) had prolonged INR, 24 (80%) had thrombocytopenia, and 17 (56.6%) were hypocoagulable by TEG. Nine of 30 (30%) had normal TEG but deranged INR and platelets. Nineteen (63.3%) cases had SIRS, 11 (36.6%) had severe sepsis, and 8 (26.6%) had bleeding. Hypocoagulable state was common in severe sepsis than sepsis/infection (81.1% versus 42.1%; *P* = 0.05) and persistent (n = 4) versus recovered SIRS (n = 15, 100% versus 33%; *P* = 0.03). Bleeders had prolonged R-time (7.8 versus 5.4 min; *P* = 0.03), smaller MA (30.2 versus 47 mm; *P* = 0.05), and α-angle (40.4 versus 62.9; *P* = 0.03) but similar INR and platelets than nonbleeders. Six patients (20%) had poor in-hospital outcomes; R-time ≥8.5 min predicted mortality with high sensitivity (83%) and specificity (100%).

**Conclusions:**

Fifth-seven percent of children with DCLD and infection were hypocoagulable by TEG. Severe sepsis and persistent SIRS worsened the coagulation status. TEG identifies bleeders better than INR and platelet count. R-time ≥8.5 min predicts a poor hospital outcome.

**What Is Known**

- Children with decompensated chronic liver disease (DCLD) often have deranged prothrombin time/international normalized ratio (PT/INR) and thrombocytopenia and were considered as auto-anticoagulated.
- Conventional coagulation tests do not predict the risk of bleeding accurately in DCLD.
- Pediatric studies assessing the role of thromboelastography (TEG) in liver disease are limited largely to transplant setting.

**What Is New**

- Nearly half of the children with decompensated chronic liver disease and infection were hypocoagulable by TEG.
- TEG identifies children with bleeding risk better than conventional coagulation test (PT/INR, platelet count).
**•** TEG can predict the outcome, with R-time ≥8.5 min predicting mortality with high accuracy.

## INTRODUCTION

The liver has an important role in hemostasis as it not only produces the coagulation factors but also the anticoagulation factors and fibrinolytic proteins (1). Chronic liver disease (CLD) patients often have a deranged international normalized ratio (INR) and/or thrombocytopenia. Traditionally, these patients were thought to be auto-anticoagulated. However, INR and platelet count do not accurately predict the risk of bleeding after invasive procedures (2). Adult studies showed a higher risk of developing thrombotic complications in cirrhotics than in the general population (3). These observations highlighted the limitations of conventional coagulation tests (CCTs) in CLD patients. Multiple studies in adults with cirrhosis have shown the superiority of thromboelastography (TEG) over CCT in assessing coagulation kinetics and guiding transfusion in bleeders or patients undergoing invasive procedures (4–6).

Sepsis affects the coagulation in cirrhotics by affecting platelet aggregation, clotting factors activation, fibrinolysis, and release of endogenous heparinoids. TEG-based studies in adults with cirrhosis and infection demonstrated a hypocoagulable state with increased bleeding risk and improvement after resolution of infection (7–9). Systemic inflammatory response syndrome (SIRS) also affects the coagulation by upregulating tissue factor and plasminogen activator inhibitor 1 and downregulation of protein C, S, and antithrombin III (10).

Pediatric studies assessing the role of TEG in liver disease are limited and available largely in the transplant setting (11). Our primary objective was to study the coagulation status of children with decompensated chronic liver disease (DCLD) and infection by TEG. The secondary objectives were to evaluate the effect of severity of infection, presence of SIRS, and resolution of infection on the coagulation status and the difference in coagulation status of children with and without bleeding manifestations.

## METHODS

The institutional ethics committee (IEC:2019-62-DM-EXP-7) approved the study and informed consent was obtained from parents of all participants before enrollment.

In this prospective pilot study conducted between April 2019 and July 2021, 30 consecutive cases admitted with DCLD and infection were enrolled. CLD was diagnosed based on clinical, imaging, and endoscopic findings (≥grade II esophageal varix) with/without liver biopsy. Standard criteria were used for etiological diagnosis of CLD and when no cause could be found, they were labeled as cryptogenic (12,13). Decompensation was defined as the presence of ascites, encephalopathy, and/or variceal bleeding.

Criteria for the diagnosis of infection included ≥1 of the following: ascitic fluid infection (polymorphonuclear count in ascitic fluid ≥250/mm^3^ and/or positive culture), bloodstream infection (BSI; positive blood culture), urinary tract infection (pyuria and ≥10^**5**^ colonies per mL of single uropathogenic organism), and pneumonia (clinical features and chest x-ray). Infection at other sites was defined by Centers for Disease Control and Prevention criteria (14). SIRS and sepsis severity were defined as per the International pediatric sepsis consensus conference definition (15). Organ failure was scored by the pediatric chronic liver failure sequential organ failure assessment (pCLIF-SOFA) (16). The Child-Pugh (17), and pCLIF-SOFA score were calculated at admission and after 7–14 days of therapy.

**FIGURE 1.**
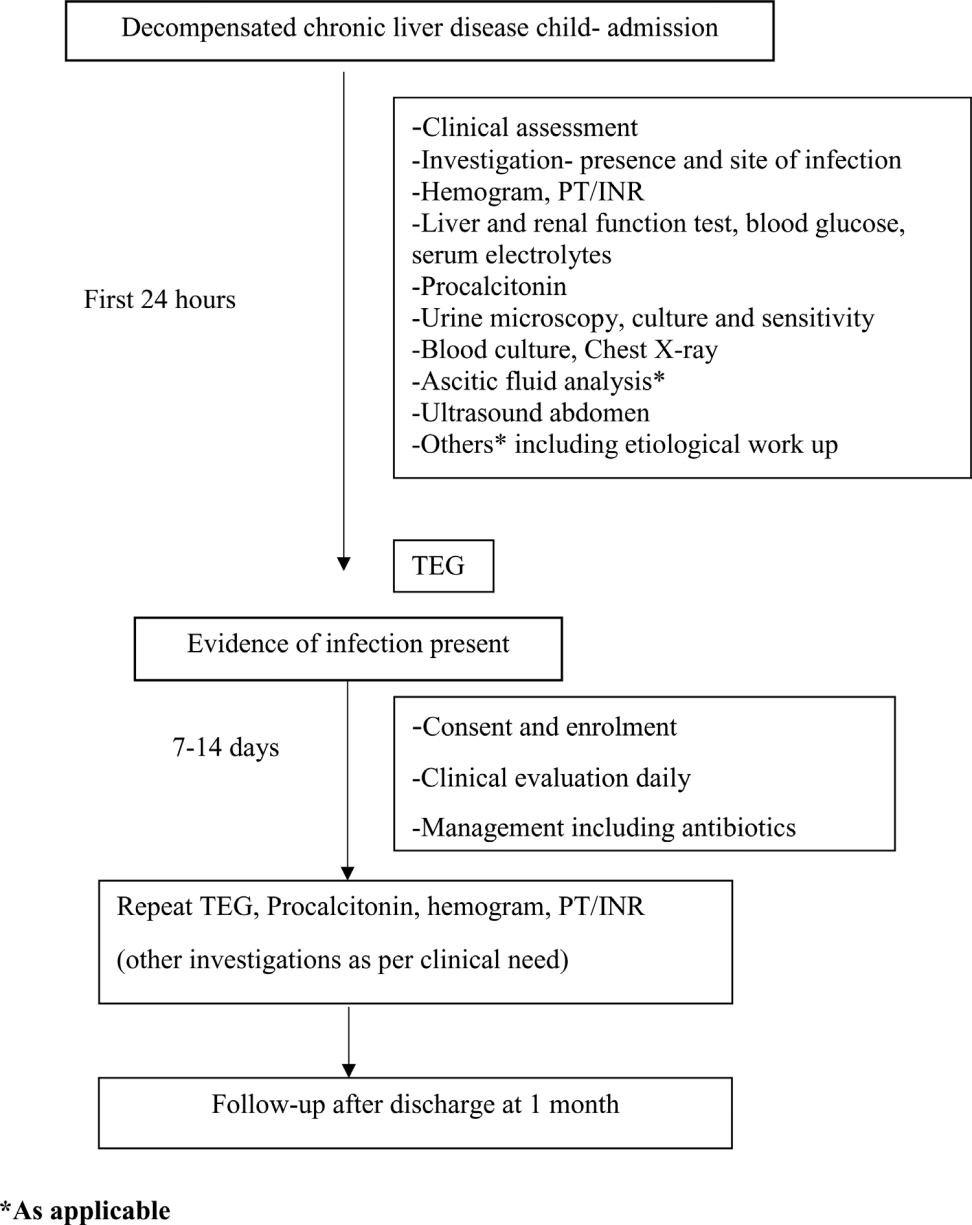
Flow chart showing study protocol for enrollment and follow-up of patients.

Figure 1 shows the study flow chart and patient enrollment. Exclusion criteria included blood product transfusion within 48 h before admission, current therapy with antiplatelet/anticoagulant medications and refusal to consent. Detailed clinical history, examination and investigations were recorded. Repeat hemogram, INR, procalcitonin (PCT), and TEG were done after 7–14 days of therapy for infection resolution. Other investigations like cultures, X-ray, and ascitic tap were repeated as per the clinical situation. Patients with clinical improvement and normal PCT in follow-up were labeled as complete resolution, those with clinical improvement but raised PCT (>0.6 ng/mL) as partial resolution and those with no clinical improvement and elevated PCT as no resolution of infection. Subjects received antibiotics as per the site of infection and culture sensitivity. Specific treatment for underlying etiology of CLD and other complications was given as per recommendations. Postdischarge follow-up was done at 1 month as out-patient.

## THROMBOELASTOGRAPHY

Whole blood (2 mL) was collected in a 3.2% sodium citrate tube and processed at the earliest and within 2 h of sampling by kaolin activated TEG (TEG 5000; Haemonetics analyzer system). The following clot formation parameters were evaluated: (a) reaction (R) time: time between the start of the test and the initial fibrin formation, (b) kinetic (K) time: time from initial fibrin formation to reach an amplitude of 20 mm, (c) alpha (α) angle: the speed at which fibrin builds up and cross-linking takes place, (d) maximum amplitude (MA): measures the ultimate strength of the fibrin clot, and (e) lysis at 30 min (LY30): percentage decline in amplitude at 30 min (18). Following clot formation parameters were measured with the coagulation index (CI). (CI = 0.1227 (R) + 0.0092(K) + 0.1655(MA) − 0.0241(α) − 5.022). Normal CI ranges from −3 to +3 (normocoagulable), <−3 represents hypocoagulable, and >3 represents hypercoagulable state (18).

## STATISTICAL ANALYSIS

Continuous variables are expressed as median with interquartile range and discrete variables as percentages. Continuous data were analyzed by the Mann–Whitney U test and paired continuous data of the same group were compared by Wilcoxon signed-rank test. Proportions were compared by chi-square/Fisher’s exact test. Receiver operating characteristics curve was drawn to calculate area under curve and cut off value (Sp+Se max). The *P* value <0.05 was considered significant. Data were analyzed by SPSS version 20.0 (IBM SPSS Statistics, Armonk, NY).

## RESULTS

Thirty children (21 boys, age 78 [15.7–180) months] with DCLD were enrolled. Of these, 29 cases had ascites, 4 had hepatic encephalopathy and 3 had variceal bleeding. Table 1 shows the demographic and clinical characteristics. Wilson disease (7, 23.3%) was the most common cause followed by autoimmune hepatitis (6, 20%). At admission, all cases had clinical evidence of infection and 27 (90%) had raised procalcitonin (2.1 [0.8–12.6] ng/mL). Severe sepsis was present in 11 (36.6%), sepsis in 9 (30%) and infection alone in 10 (33.3%) cases. Spontaneous bacterial peritonitis (n = 19, 63.3%) was the commonest site of infection followed by pneumonia (Table 1).

**TABLE 1.** Demographic and clinical characteristics at admission

Variables	N = 30
Age (in months)	78 (15.7–180)
Gender (male:female)	21:9
Weight (z score)	–1.4 (–2.9 to –0.6)
Height (z score)	–1.5 (–3.0 to –0.3)
First decompensation/ previous decompensation (n)	19/11
Etiology of chronic liver disease, n (%)	
Wilson disease	7 (23.3)
Autoimmune hepatitis	6 (20)
Biliary atresia	5 (16.7)
Galactosemia	3 (10)
Secondary biliary cirrhosis	2 (6.7)
Cryptogenic	2 (6.7)
BASD, PFIC, BCS, tyrosinemia, secondary hemochromatosis	1 (3.3) case each
Site of infection, n (%)	
Spontaneous bacterial peritonitis	19 (63.3)
Pneumonia	6 (20)
Cholangitis	4 (13.3)
Meningitis	2 (6)
Bacteremia	2 (6)
Urinary tract infection	1 (3.3)
No focus identified	3 (10)
Site of bleed (n = 8)	
Variceal, n (%)	3 (37.5)
GI mucosal, n (%)	3 (37.5)
Epistaxis and Pulmonary hemorrhage, n (%)	1 (12.5)
Skin hematoma/ooze from paracentesis site, n (%)	1 (12.5)
CTP score	11 (11–13)
PELD score (n = 21)	29 (22–33)
MELD score (n = 9)	30 (25–32)
pCLIF-SOFA score	7 (6–8)
Total bilirubin (mg%)	10.1 (3.0–17.4)
SGOT (IU/L)	102.5 (56–222.2)
SGPT (IU/L)	73 (43.8–186.5)
Albumin (g/dL)	2.6 (2.1–2.8)
Alkaline phosphatase (IU/L)	248 (133.3–556.5)
Gamma-glutamyl transpeptidase (IU/L)	66.5 (32.3–148)
S. creatinine (mg/dL)	0.5 (0.3–0.6)
S. sodium (mEq/L)	134 (128–137)
Hospital mortality, n (%)	6/30 (20%)

All continuous values are expressed as median (IQR). BASD = bile acid synthetic defect; BCS = Budd-chiari syndrome; CTP = Child-Turcotte-Pugh; MELD = model for end-stage liver disease; pCLIF-SOFA = pediatric chronic liver failure sequential organ failure assessment score; PELD = pediatric end-stage liver disease; PFIC = progressive familial intrahepatic cholestasis; SGOT = serum glutamic-oxaloacetic transaminase; SGPT = serum glutamic-pyruvic transaminase; TEG = thromboelastography.

### Baseline TEG Parameters and Relation to INR/Platelet Count

Of the 30 children, 29 (96.7%) had deranged INR (>1.5) and 24 (80%) had thrombocytopenia (<150 000/mm^3^). Seventeen (56.6%) were hypocoagulable (CI: −5.9 [−9.4 to −4]) and 13 (43.4%) had normal coagulation by TEG. The individual parameters in these 17 cases were as follows: R- 6.8 (5.2–8.4) min, K- 4.8 (3.1–7.4) min, MA- 35.3 (28.9–41.4) mm, α-angle- 46°(35.4–54.0), LY30- 0 (0–1.5). Of the 17 children with hypocoagulable TEG, one had normal INR (5.8%) and 2 (11.8%) had normal platelet count. Of the 13 children with normocoagulable TEG, 13 (100%) had deranged INR and 9 (69.2%) had thrombocytopenia. Thus, 9 of 30 (30%) DCLD children had deranged INR and thrombocytopenia but normal TEG. There was a significant difference in INR and platelet count (per mm^3^) of patients with hypocoagulable TEG (n = 17) versus normocoagulable TEG (n = 13) (3.4 [2.5–4.6] versus 2.1 [1.9–3.0]; *P* = 0.09) and (57 000 [46 500–98 500]/mm^3^ versus 120 000 [75 500–157 000]/mm^3^; *P* = 0.03), respectively.

### TEG Parameters and SIRS

SIRS was present in 19 (63.3%) cases at admission. Subjects with SIRS were more often hypocoagulable (63.2% versus 45.5%) than no SIRS, but the difference was not significant. (Table 2) With treatment, SIRS persisted in 4 (13.3%) and resolved in 15 (50%) cases. All patients with persistent SIRS were hypocoagulable as compared to only 33.3% of those with resolved SIRS (*P* = 0.03). Persistent SIRS was associated with poor coagulation status with significantly lower MA, α-angle, and LY30 in comparison to resolved SIRS (Table 2).

**TABLE 2.** Effect of systemic inflammatory response syndrome on thromboelastography parameters at admission and follow-up

	Admission			Follow-up		
Parameter	SIRS (n = 19)	No SIRS (n = 11)	*P*	SIRS recovered (n = 15)	SIRS persistent (n = 4)	*P*
R (min)	5.7 (4.9–7.1)	5.8 (5.2–6.8)	0.74	5.5 (4.8–8)	7.4 (4.5–16.1)	0.39
K (min)	2.4 (1.6–4.8)	3.7 (1.8–8.3)	0.83	2.3 (1.7–3.5)	4.9 (4.2)	0.11
MA (mm)	43.7 (35.3–56.8)	42.5 (26.7–52.7)	0.46	47.9 (38.4–61.1)	26.5 (12.2–39.9)	0.02
Alpha angle (^o^)	56.7 (46–66.5)	48.3 (34.5–65.2)	0.74	63 (44.7–66.3)	32.1 (14.5–44.7)	0.02
CI	–4 (–6 to 0.8)	–1.6 (–9.2 to 0)	0.83	–1.2 (–5.7 to 0.4)	–5.6 (–8.2 to –4.6)	0.07
LY30	0.7 (0–1.1)	0 (0 to 1.6)	0.37	0.7 (0–18)	0	0.03
Hypocoagulable*	12 (63.2)	5 (45.4)	0.34	5 (33.3)	4 (100)	0.03
Normocoagulable*	7 (36.8)	6 (54.5)		9 (60)	0	
Hypercoagulable*	0	0		1 (6.6)	0	
INR	2.9 (2.1–4.7)	2.3 (2.1–2.4)	0.31	2 (1.3–2.3)	7 (3.1–9.3)	0.01
Platelet/mm^3^ ×10^3^	72 (50–130)	97 (47–164)	0.53	100 (60*–*157)	59.5 (45.5*–*165)	0.48

All variables are expressed in median (IQR) except

*n (%) as proportions. CI = coagulation index; CTP = Child-Turcotte-Pugh score; INR = international normalized ratio; K = kinetic time; LY30 = lysis at 30 min; MA = maximum amplitude; R = reaction time.

### Severity of Sepsis and TEG Parameters

Patients with severe sepsis were more often hypocoagulable than those with infection/sepsis (9/11 versus 8/19; *P* = 0.05). After treatment, 15 cases had complete resolution, 10 had no resolution and 5 had partial resolution of infection. Subjects with complete resolution of infection had better R time (5.4 [3.8–5.6] versus 7.4 [4.5–9.9] min, *P* = 0.1), LY30 (0.7 [0–1.2] versus 0 [0–0.7], *P* = 0.08) and less hypocoagulable state (40% versus 60%; *P* = 0.4) than those with persistent infection (Supplemental Digital Content Table 1, http://links.lww.com/PG9/A112), but the difference was not statistically significant. They also had a significantly better INR (2.0 [1.4–2.4] versus 4.2 [2.0–6.8]; *P* = 0.04) than those with persistent infection. Supplemental Digital Content Figure 1 (http://links.lww.com/PG9/A113) shows the representative TEG tracings of improvement in a case with resolution of infection (A and B) and worsening in a child with persistent infection despite therapy (C and D).

### Relation of TEG Parameters With Bleeding

**FIGURE 2.**
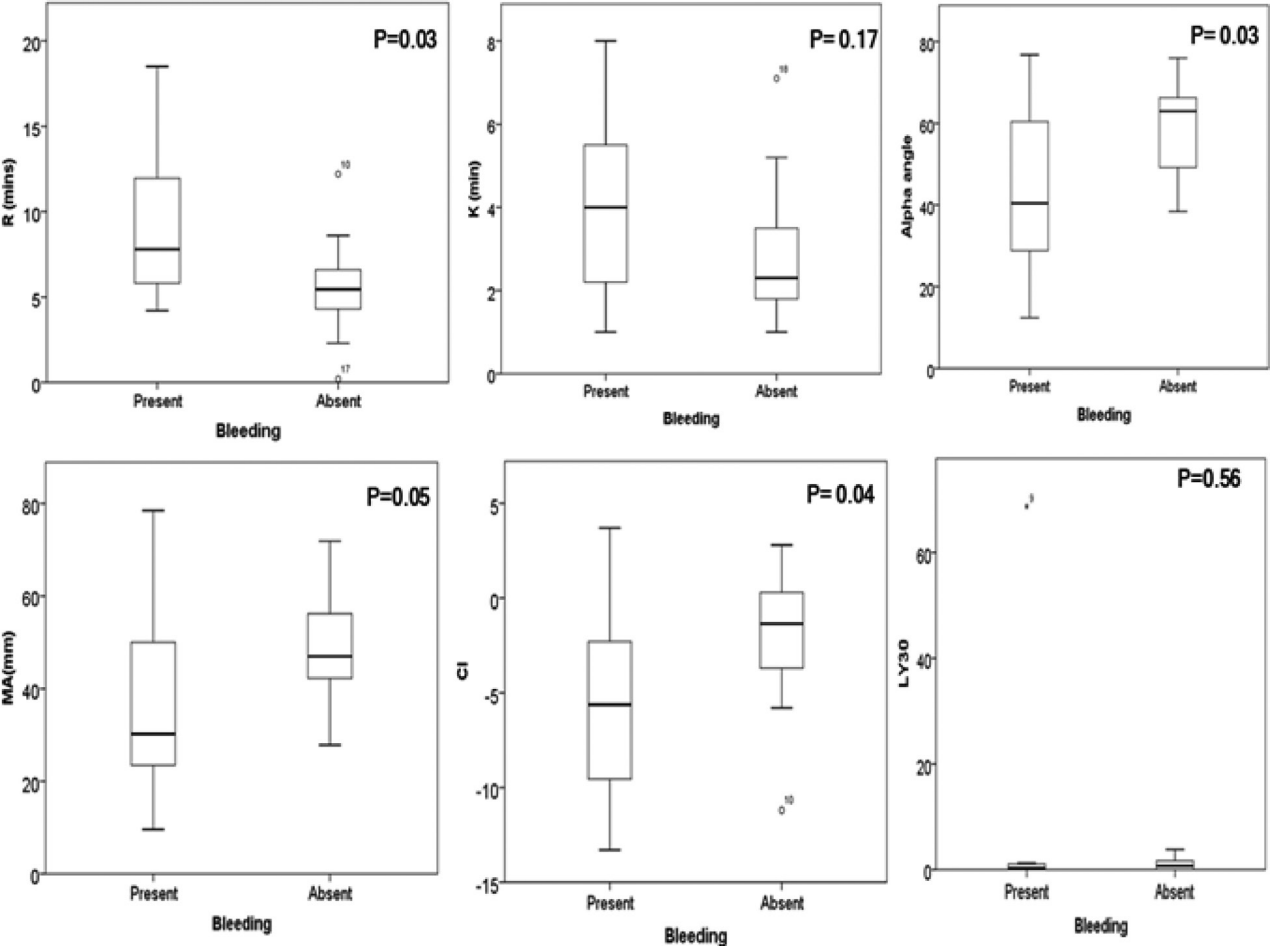
Comparison of coagulation factors by thromboelastography (TEG) variables in cases with and without bleeding manifestation.

Eight (26.6%) children had bleeding manifestation (major-5, minor-3); the site of bleeding is given in Table 1. All cases with major bleed required blood transfusion and 2 required fresh frozen plasma. A higher proportion of bleeders had hypocoagulability on TEG (6 [75%] versus 7 [31.8%]; *P* = 0.04) than nonbleeders. Patients with bleeding manifestation had significantly higher R time, lower MA, α-angle, and CI (Fig. 2). There was no significant difference in the proportion of patients with deranged INR (INR ≥ 1.5) between bleeders and nonbleeders (7/8 versus 21/22; *P* = 0.46). Equal proportion of bleeders and nonbleeders had thrombocytopenia (8/8 versus 17/22; *P* = 0.28) and severe thrombocytopenia (platelet < 50 000/mm^3^, 3/8 versus 4/22; *P* = 0.61). CCT (INR and platelet count) were not significantly different between bleeders and nonbleeders.

### Severity of Liver Dysfunction and TEG

At baseline, the PELD (28 [14–29] versus 31 [22–38]; *P* = 0.28), MELD (25 [24–30] versus 32 [27–32]; *P* = 0.93), CTP (12 [11–12] versus 12 [12–13]; *P* = 0.6), and pCLIF-SOFA score (7 [6–8] versus 7 [6–9]; *P* = 0.8) were similar between patients with normocoagulable (n = 13) and hypocoagulable (n = 17) TEG. On follow-up, those with hypocoagulable TEG (n = 13) had higher PELD (36 [25–42] versus 24 [6–26]; *P* = 0.01), CTP (11 [10–13] versus 10 [9–11]; *P* = 0.08), and pCLIF-SOFA (7 [6–9] versus 6 [5–7]; *P* = 0.02) than subjects with normocoagulable TEG (n = 16). One case is hypercoagulable. Subjects with bleeding manifestation had higher PELD (36 [3–51] versus 25 [16–30], *P* = 0.38), MELD (42 [33–42] versus 27 [22–30], *P* = 0.06), CTP (12 [7–13] versus 10 [9–11], *P* = 0.12), and PCLIF-SOFA (9 [3–10] versus 6 [5–7], *P* = 0.09) than nonbleeder but not significantly.

### Comparison of Poor Versus Good Hospital Outcome

None of these children could be offered liver transplantation due to logistic reasons. Six (20%) children died after 24 (13–27.5) days of hospitalization due to end-stage liver disease with encephalopathy (n = 4), 2 cases also had uncontrolled sepsis with DIC, pulmonary hemorrhage (n = 1), and refractory EVL ulcer bleed (n = 1). Of the 24 children in follow-up, 3 died (3/24, 12.5%) within 28 days. Overall mortality was 30% (9/30) at 1-month postdischarge.

R-time was significantly prolonged in patients with poor outcome as compared to survivors at admission and in follow-up (Supplemental Digital Content Table 2, http://links.lww.com/PG9/A112). Other parameters like K time, MA, α-angle, and LY30 were more affected in nonsurvivors than survivors both at admission and in follow-up but the difference was not significant (Supplemental Digital Content Table 2, http://links.lww.com/PG9/A112 and Supplemental Digital Content Figure 2, http://links.lww.com/PG9/A113). R time of ≥8.5 min at follow-up predicted poor hospital outcome with a sensitivity of 83% and specificity of 100%, with an area under the curve of 0.986 (95% CI [0.951–1], *P* = 0.000) (Supplemental Digital Content Figure 3, http://links.lww.com/PG9/A113).

Furthermore, nonsurvivors had significant worsening of R, MA, α-angle and nonsignificant deterioration of K time, INR, and platelets despite therapy during the hospital stay (Supplemental Digital Content Table 2, http://links.lww.com/PG9/A112). In contrast, survivors showed improvement in all coagulation parameters which was significant for K time, α-angle, CI, INR, and platelets.

Apart from coagulation abnormalities, nonsurvivors had higher CTP (13 [12–13] versus 11 [10–12]; *P* = 0.01), pCLIF-SOFA score (9 [7–10] versus 7 [6–8]; *P* = 0.04) and more severe sepsis (66.6% versus 29.1%; *P* = 0.03). There was no significant difference in the frequency of hepatic encephalopathy (16.6% versus 16.6%; *P* = 1), bleeding (50% versus 20.8%; *P* = 0.3) and SIRS (83.3% versus 58.3%; *P* = 0.3) between the 2 groups. As the number of cases was small, we could not perform multivariate analysis for identifying factors predicting in-hospital outcome.

## DISCUSSION

Our prospective study provides insight into the coagulation status of children with DCLD and sepsis. 57% of the cases were hypocoagulable by TEG at admission. There is no comparable pediatric data; however, our figure is comparable to the adult data. Blasi et al studied adults with ACLF and DCLD and found that 61% and 29%, respectively, had hypocoagulability by rotational thromboelastometry (ROTEM). A third of ACLF group had infection as precipitant (19). Premkumar et al showed that 57 % of adults with ACLF and sepsis had hypocoagulability by TEG (20). In our study, relying on INR and platelets, labeled 30% of DCLD cases with normal TEG to have abnormal coagulation. TEG was superior to INR/platelet for assessment of coagulation. This is similar to a prospective analysis of acutely ill adults with CLD, which showed an inconsistent correlation with CCT (21).

### Thromboelastographic Profile Changes in DCLD With SIRS/Sepsis

SIRS was present in 63.3% of our cases at admission, it persisted in 13.3% despite therapy and resolved in 50%. Hypocoagulable state at admission was more common in those with SIRS, but not statistically significant. This could be because of a small sample size. However, all patients with persistent SIRS were hypocoagulable as compared to those with resolved SIRS. Persistent SIRS was associated with lower MA, α-angle and LY30 when compared with resolved SIRS. Association of persistent SIRS with a hypocoagulable TEG profile was shown by Blasi et al (19). They demonstrated that hypocoagulability correlated with ongoing systemic inflammation and a higher 28- and 90-day mortality. Hypocoagulability in ongoing SIRS could be due to exhaustion of clotting factors, fibrin formation, activation of clot lysis and platelet dysfunction as demonstrated by a decrease in α-angle and MA in our study (22). In addition, the mortality rate with persistent SIRS was twice as compared to resolved SIRS (50% [n = 2/4] versus 20% [n = 3/15]; *P* = 0.27) and 5 times that of no SIRS (n = 1/11 [9.1%] versus 2/4 [50%]; *P* = 0.15). The lack of statistical significance may be due to small number of cases and needs further evaluation. Premkumar et al demonstrated that ACLF subjects with SIRS/sepsis had worse coagulation status (prolonged R and K time, lower MA and α-angle) indicating lower clot stability and thrombocytopenia (20).

### TEG Parameters and Bleeding Events

Children with bleeding had poorer coagulation status, as evidenced by longer R-time suggesting defective clot formation, smaller MA, and α-angle (consistent with lower clot stability) than nonbleeders. Chau et al studied adult cirrhotics with variceal bleed and compared TEG parameters in those with and without rebleed. The rebleeding group was more hypocoagulable, with significantly longer R-time, K-time, and smaller α-angle when compared with the nonrebleeding group. CCT showed no significant difference between the 2 groups, similar to our observation (23). De Pietri et al showed that the TEG-guided transfusion strategy was superior to CCT-guided transfusion, it reduced the use of blood products without any increase in bleeding complications in cirrhotic adults before invasive procedures (24). Hence, in DCLD cases with bleeding, TEG is preferred over CCT for evaluation. However, TEG has its limitation and is likely to underestimate the hemostatic potential in liver disease, as it does not account for von Willebrand factor and protein C levels (25). It is possible that in a proportion of these sick children with hypocoagulability on TEG, there is hemostatic “rebalance” as shown in adults (26). Nearly, 40% (3/8) of bleed was PHT-related and bleeders had higher PELD/MELD, CTP and PCLIF-SOFA than nonbleeder in our study. This suggests that poorer synthetic liver function, severity of PHT, sepsis, and organ failure collectively contribute to the higher bleeding risk in these sick cases (25).

### Severity of Infection and Its Impact on Coagulation Profile

With regards to the infection severity and its impact, children with severe sepsis were more often hypocoagulable than those with infection/sepsis alone. Similar results were shown by Zhou et al, adults with septic shock had worse CI than those with sepsis alone (−5.6 ± 6.5 versus −1.3 ± 3.7; *P* = 0.019) (27).

### Sepsis Resolution and Coagulation Profile

In our study, we did not find any significant difference in TEG parameters except for longer R time in those with resolved vs persistent infection after therapy. CI and proportion of hypocoagulable cases were lower in resolved versus persistent infection but not significantly different. On comparing baseline and follow-up TEG parameters, CI showed improvement in cases with resolved infection and worsening in those with persistent infection (statistically insignificant). Papatheodoridis et al reported significant improvement after 5 days of infection treatment in R-time, K-time, α-angle, MA, and PT in adults with DCLD and infection, while R-time, K-time, and α-angle significantly worsened in those with persistent infection (9). The lack of significance in our study could be due to smaller sample size.

This highlights the importance of early identification and aggressive management of sepsis in cirrhotics. Infection is an important contributor of poor outcome in adults with cirrhosis. In a systematic review, 40.4% patients with infection and 19.5% without infection (*P* = 0.00001) died during the 3-month follow-up after discharge (28). This is similar to the poor outcome in 30% of our children with DCLD and infection.

### Association of Mortality and Coagulation Failure in Children With DCLD

We found that R ≥8.5 min at follow-up predicted mortality with high sensitivity and specificity, similar to observation of Premkumar et al in adults with ACLF (R-time >9 minutes and MA < 18 mm predicted major bleeding and mortality) (20). Zhe-Zhu et al studied adults with hepatitis B-related ACLF and found significantly higher K time, lower α-angle, MA, CI, and LY30 in subjects with poor outcomes. A combination of MA and INR predicted mortality (29). Goyal et al demonstrated that INR, LY30 and MA predicted mortality in adults with ACLF and DCLD (30). All these studies suggest that TEG parameters are more deranged in patients with poor outcome. The difference in the specific parameter predicting mortality could be due to differences in study population (ACLF versus DCLD, etiology, sepsis, other systemic diseases, age, etc). Saini et al evaluated children with sepsis without liver disease and found that longer K and R time and lower MA were associated with worsening sepsis and ongoing inflammation (31). These findings are in tandem with our observations.

Children with poorer liver function and organ failure (higher PELD/MELD, CTP, pCLIF-SOFA) were more likely to have persistent hypocoagulable state despite treatment, bleeding, and poor outcome. This suggests that the abnormal TEG is not the cause of poorer outcome but a reflection of the sicker state of these cases secondary to more severe liver disease and sepsis. The association of longer R-time with poor outcome is likely explained by severity of underlying liver disease as the R-time is directly proportional to concentrations of coagulation factors, and thus to synthetic capacity of the liver. As the changes in TEG due to ACLF and sepsis with normal liver resemble each other (26), it is not possible for us to dissect the relative contribution of these factors as all our cases had DCLD and infection.

Furthermore, we observed progressive worsening of TEG parameters with time in nonsurvivors, and improvement in survivors. This suggests that TEG can be an additional parameter for monitoring of DCLD patients with infection, as worsening suggests nonresponse and poor outcome.

The strength of this study is that it is one of the few prospectively conducted studies for assessing hemostatic dysfunction in children with DCLD and infection using TEG. But it has some important limitations. First, we have used adult reference value for calculating the coagulation index (CI) and defining hypocoagulability and hypercoagulability rather than age specific pediatric references due to non-availability. Other limitations include a small sample size, lack of correlation with parameters like aPTT, fibrinogen, D-dimer, protein C and S levels, and the absence of a comparative arm of DCLD cases without infection.

In conclusion, we have shown that 57% of children with DCLD and infection were hypocoagulable by TEG. Coagulation status is more deranged in patients with bleeding, severe sepsis, persistent SIRS and infection despite therapy. TEG is superior to INR/platelets for identifying bleeders. R ≥ 8.5 min at follow-up predicts poor outcome with high accuracy. Our study highlights the need of comprehensive coagulation studies in larger number of DCLD children.

## ACKNOWLEDGMENTS

Written informed consent was taken from parents of all participating subjects before enrollment.

## Supplementary Material
