# Supplementary material for: Hypocoagulability in Children With Decompensated Chronic Liver Disease and Sepsis: Assessment by Thromboelastography
Source: JPGN Rep. 2023 Jun 9;4(3):e324. doi: 10.1097/PG9.0000000000000324 (PMC10435032; doi:10.1097/PG9.0000000000000324)
Supplement: Supplementary file 1 [file pg9-4-e324-s001.pdf]

Supplemental Digital Content 1

Supplementary Table 1: Comparison of thromboelastography variables in subjects with resolution vs. persistence of infection in follow-up.

Parameter	**Infection resolved (n = 15)**	**Infection not resolved (n = 10)**	**P**
R (min)	*5.4 (3.8-5.6)*	*7.4 (4.5-10.0)*	*0.1*
K (min)	2.3 (1.7-4)	2.4 (1.4-4.9)	0.82
MA (mm)	45.9 (38.4-55.8)	42.4 (25.8-56.5)	0.25
Alpha angle (º)	60.5 (45.3-66.3)	53.6 (37.5-71.8)	0.54
CI	-1.7 (-4.4 to 0.4)	-4.4 (-6.7 to -0.2)	0.28
LY30	*0.7 (0 to 1.2)*	*0 (0 to 0.7)*	*0.08*
Hypocoagulable*	6 (40)	6 (60)	0.42
Normocoagulable*	8 (53.3)	4 (40)	
Hypercoagulable*	1 (6.7)	0	
INR	*2.0 (1.4-2.4)*	*4.2 (2.0-6.8)*	*0.04*
Platelets /mm^3 x10^3	89 (59-127)	161.5 (55.2-247.5)	0.1

*All variables are expressed in median (IQR) except * n (%) as proportions,* ***CI:*** *Coagulation index;* ***INR****- International normalised ratio****; K:*** *kinetic time,* ***LY30:*** *lysis at 30 min;* ***min****: minutes;* ***MA:*** *maximum amplitude;* ***R****: reaction time*

Supplementary Table 2: Comparison of coagulation parameters in survivors and non-survivors at admission and follow-up in hospital

Parameter	Admission			Follow up		
	**(A) Survived (n = 24)**	**(B) Not survived (n = 6)**	**P^**	**(C) Survived (n = 24)**	**(D) Not survived (n = 6)**	**P$**
R (min)	*5.6 (4.9-6.8)*	*7.4 (6.5 to 10.1)*	*0.01*	*5.4 (4.2 to 6.1)*	*10.7(8.3 to 15.7) #*	*<0.0001*
K (min)	2.4(1.8-5.7)	3.4 (1.4-4.7)	0.67	2.3 (1.8 to 3.9) *	3.8 (1.5 to 5.4)	0.55
MA (mm)	44.9 (31.1-56.7)	37.8 (32-53.4)	0.48	46 (42.2 to 55.7)	27.6 (17.4 to 57.4) #	0.12
Alpha angle (°)	55.3(45.4-65.1)	51.8 (42-69.1)	0.98	61.5(47.4 to 66.3) *	39.9 (18.8 to 65.5) #	0.08
CI	-3.4 (-6.0 to -0.2)	-5.0 (-9.1 to 1.2)	0.64	*-1.5 (-4.2 to 0.4) **	*-7.5 (-11.7 to -1.7)*	***0.01***
LY30	0.3 (0 to 1.1)	0.4 (0 to 2.6)	0.95	0.7 (0 to 1.6)	0 (0 to 18)	0.36
Hypocoagulable$	13 (54.2)	4 (66.6)	0.67	9 (37.5)	4 (66.6)	0.35
Normocoagulable$	11 (45.8)	2 (33.3)		14 (58.3)	2 (33.3)	
Hypercoagulable$	0	0		1 (4.2)	0	
INR	2.8 (2.2-3.5)	3.8 (1.9-6.2)	0.66	2.0 (1.6-2.6) *	4.2 (1.9-9.1)	0.08
Platelet count/mm³ x10³	78 (47.7-132)	104 (56.5- 402.2)	0.33	110 (67.5-155) *	50.5 (38.7- 250.5)	0.28

All variables are expressed in median (IQR) except $ as n (%) as proportions,

***CI:** Coagulation index; **INR:** international normalised ratio; **K:** kinetic time; **LY30**: lysis at 30; **min;** minutes; **MA:** maximum amplitude; **R:** reaction time. * Significant difference in coagulation test at admission and follow-up in survivors (between A and C, n-24), # Significant difference in coagulation test at admission and follow-up in non-survivors (between B and D, n-6). ^shows difference in parameters in columns A and B $ shows difference in parameters in columns C and D*
