# Supplementary material for: Hypocoagulability in Children With Decompensated Chronic Liver Disease and Sepsis: Assessment by Thromboelastography
Source: JPGN Rep. 2023 Jun 9;4(3):e324. doi: 10.1097/PG9.0000000000000324 (PMC10435032; doi:10.1097/PG9.0000000000000324)

Supplemental Digital Content 2

Supplementary Figure 1: Changes in Thromboelastography (TEG) parameters in children with cirrhosis with infection at admission and in follow-up

(A) At baseline showing prolonged reaction time R – 10.7 min, clot formation time K-18.8 min, α-angle of 15.3°, maximum amplitude MA 22.1 mm.

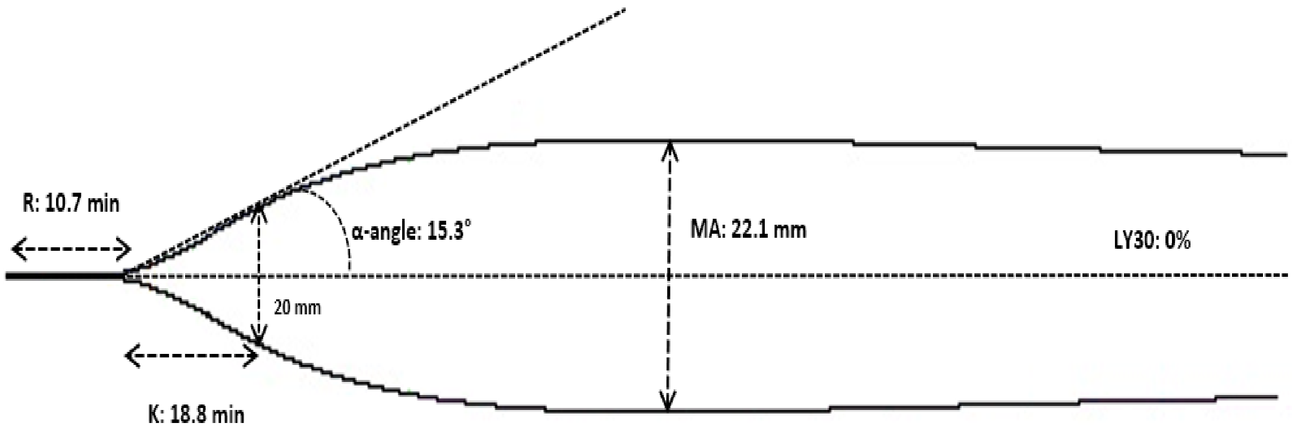

(**B**) TEG tracing in the same child shows normalisation of R - 4.6 min, K – 2.4 min, MA-50.2mm, α-angle of 49.2° after sepsis resolution

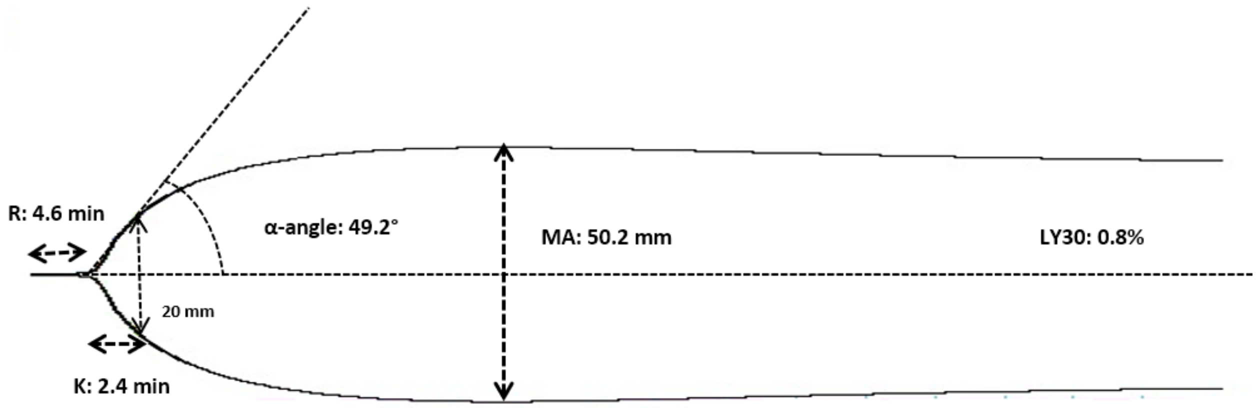

(C) TEG tracing at admission in a decompensated biliary atresia with sepsis shows baseline TEG parameters R-7.1 min, K-4.3 min α-angle of 46°, MA -37.8 mm, LY30 - 0%

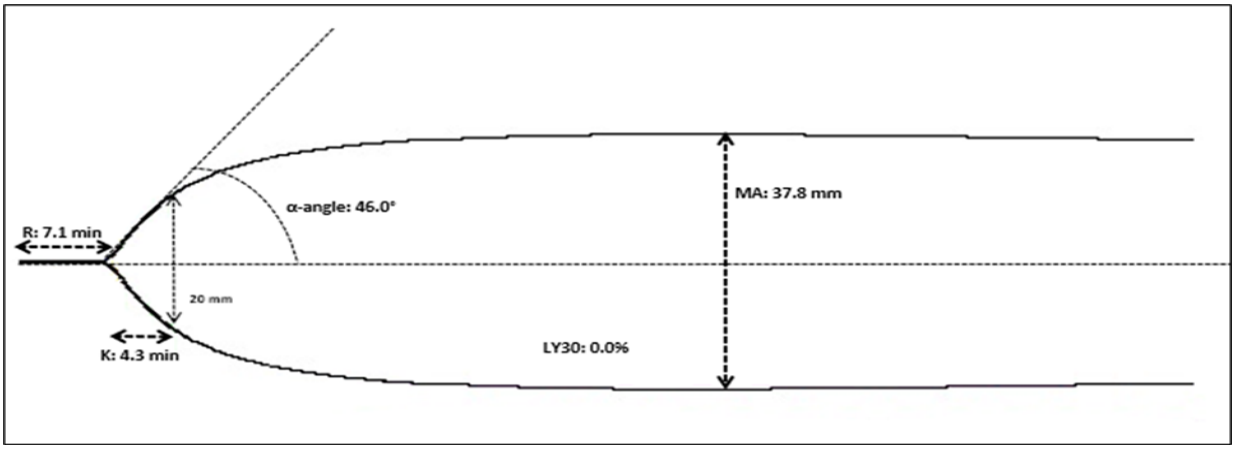

(D) in same child shows further worsening of R- 14.7 min, K-5.5 min, reduced MA-27.4 mm and α-angle-36.7° and markedly increased LY30 (68.7%) suggesting hyperfibrinolysis after worsening of sepsis during hospital stay

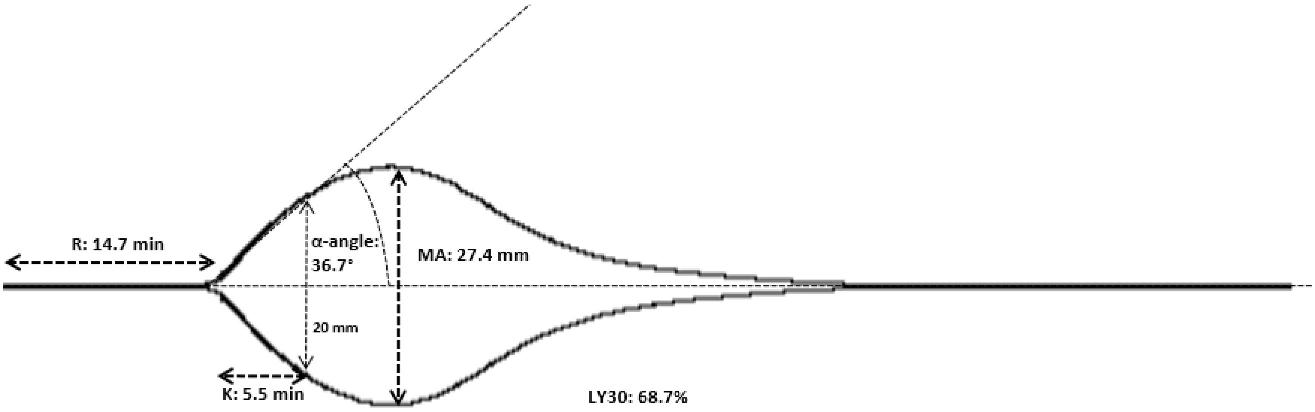

Supplementary Figure 2: Trend of Thromboelastography (TEG) parameters showing progressive worsening in non-survivors and improvement in survivors at admission and in follow-up.

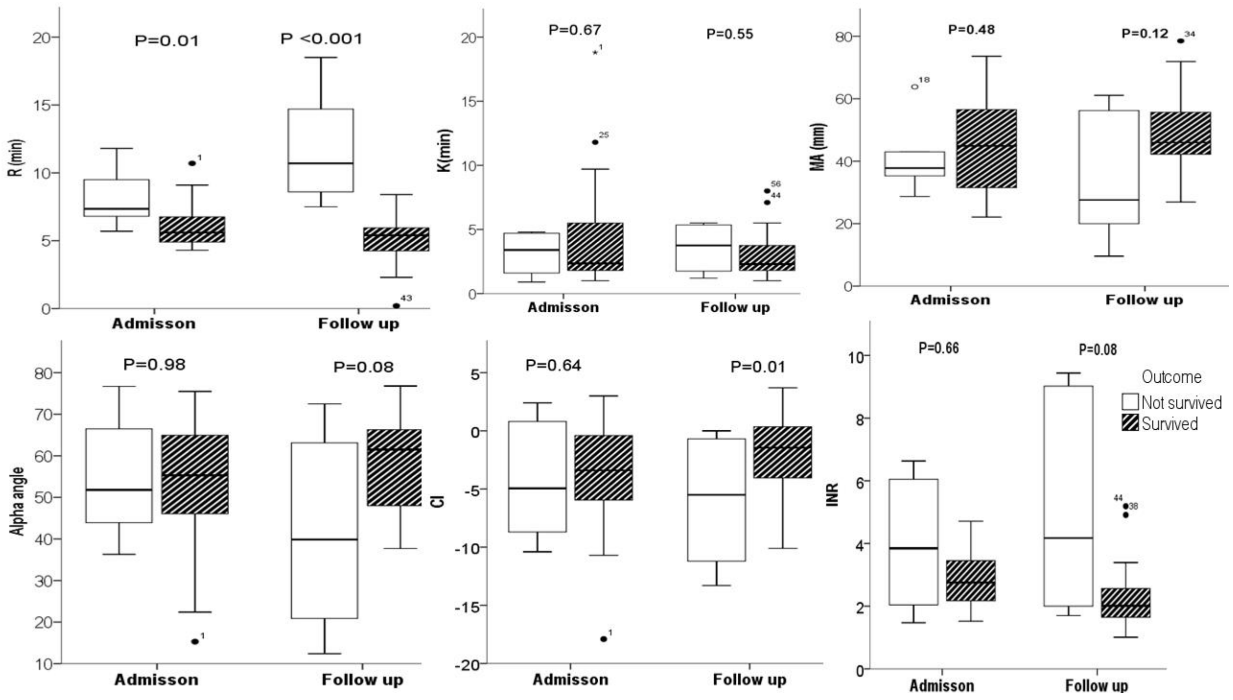

Supplementary Figure 3: Receiver operating characteristic curve of R time (at follow up) for predicting poor hospital outcome

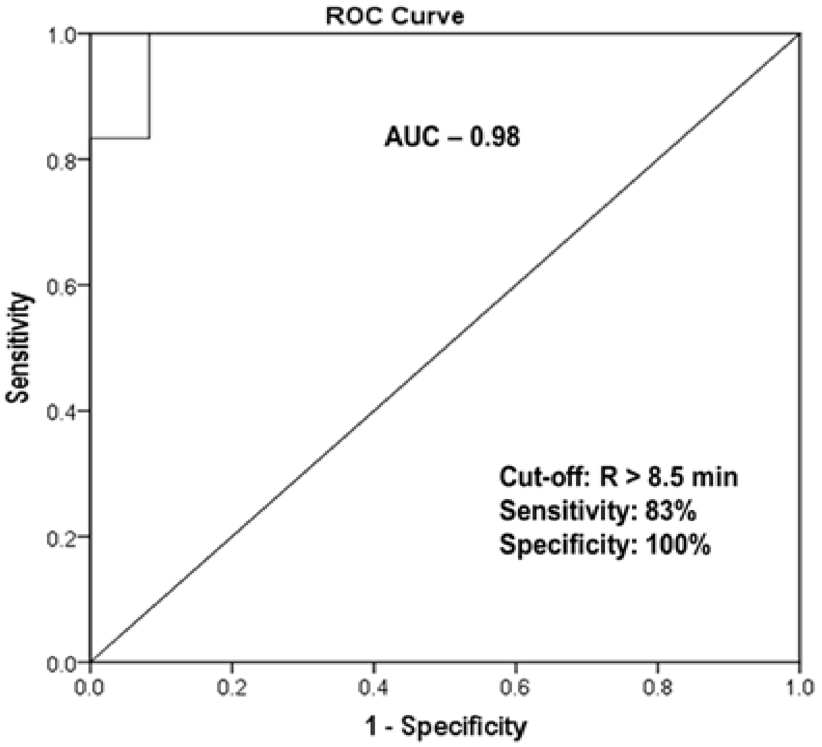

Supplement: Supplementary file 2 [file pg9-4-e324-s002.pdf]
